# Delayed postoperative spinal epidural hematoma after one-hole split endoscope discectomy: a case report and literature review

**DOI:** 10.3389/fsurg.2026.1737628

**Published:** 2026-02-25

**Authors:** Haonan Li, Youzhi Zhou, Yubo Zhou, Tao Liu, Peng Gao, Miao Ge, Xu Zhong, Koji Uotani, Masato Tanaka, Ying Tan, Mishan Wu

**Affiliations:** 1 Faculty of Chinese Medicine, Macau University of Science and Technology, Taipa, Macao SAR, China; 2 Department of Spinal Surgery, Weifang Hospital of Traditional Chinese Medicine, Shandong Second Medical University, Weifang, China; 3 First Clinical Medical College, Shandong University of Traditional Chinese Medicine, Jinan, China; 4 Department of Orthopaedic Surgery, Okayama University Hospital, Okayama, Japan

**Keywords:** lumbar discectomy, minimally invasive spine surgery, one-hole split endoscopy, postoperative spinal epidural hematoma, spine

## Abstract

**Background:**

One-Hole Split Endoscopic (OSE) discectomy is an emerging minimally invasive technique for lumbar degenerative disease. While OSE offers advantages such as reduced tissue dissection, it is not exempt from complications inherent to spinal surgery. Postoperative spinal epidural hematoma (POSEH), though rare, is a serious complication that can lead to significant neurological deterioration if not managed promptly. Although POSEH has been documented with other endoscopic lumbar procedures, no cases of delayed POSEH (DPOSEH) following OSE have been reported in the literature to date.

**Case presentation:**

A 69-year-old male underwent OSE discectomy at L4–L5 for symptomatic disc herniation. The initial postoperative course was uneventful, with improvement in radicular symptoms and intact neurological function. However, on postoperative day 5, he developed acute back pain, bilateral lower limb weakness, saddle anesthesia, and fecal incontinence. Emergency MRI confirmed a compressive epidural hematoma extending from L4 to L5. During the urgent surgical evacuation, multiple organized blood clots of varying sizes were identified and removed. Through postoperative rehabilitation therapy, the patient's left lower limb muscle strength gradually improved, though bowel and bladder dysfunction persisted.

**Conclusions:**

The present case highlights that, despite the absence of prior literature on this complication in OSE, the risk of symptomatic epidural hematoma exists similarly to other endoscopic spinal techniques. Early recognition, prompt imaging, and immediate surgical intervention are critical to optimizing neurological recovery. Surgeons should maintain a high index of suspicion for POSEH in OSE patients presenting with acute neurological decline, even beyond the typical 72-h postoperative window.

## Introduction

One-Hole Split Endoscopic (OSE) Discectomy is a recent advancement in minimally invasive spine surgery (MISS) for Lumbar degenerative disease. It was first reported and clinically applied in 2019 (1). The OSE technique has evolved from the established Unilateral Biportal Endoscopy (UBE) system. While both utilize two distinct channels for endoscopy and instrumentation, OSE uniquely consolidates these pathways into a single operative portal via one minimal incision, thereby minimizing tissue dissection while preserving the ergonomic benefits of biportal surgery (1). This single-incision, split-channel design enables the endoscope and instruments to move freely in parallel, eliminating the triangular “V-angle” limitation of biportal systems. Consequently, OSE can achieve a wider field of view with fewer blind spots, theoretically reducing the risk of neural tissue injury while maintaining effective decompression (2). Over the past few years, OSE has been applied to treat lumbar degenerative diseases as an alternative to UBE. Both UBE and OSE share the advantages of endoscopic surgery—such as less muscle trauma and faster rehabilitation—while aiming to preserve spinal stability by avoiding extensive bone resection.

Despite the demonstrated advantages and expanding indications of these techniques, it is imperative for surgeons to recognize and manage their uncommon but clinically significant complications. Postoperative spinal epidural hematoma (POSEH) is one such uncommon yet potentially devastating complication associated with spine surgeries. POSEH refers to an accumulation of blood in the epidural space after an operation, which can compress the dural sac or nerve roots. It is fortunately rare in the era of MISS, but when it occurs, it may lead to acute neurological deficits if not recognized and treated promptly (3).Clinicians differentiate acute onset cases from delayed onset cases using a threshold of about 72 h postoperatively (4). Acute POSEH typically manifests within the first day (5–8). Delayed POSEH is relatively uncommon but does occur. The incidence of delayed-onset hematomas was significantly lower than that of early-onset cases, with one pooled analysis reporting rates of 0.16% vs. 0.41%, respectively (9).

To the best of our knowledge, no cases of delayed postoperative spinal epidural hematoma (DPOSEH) following OSE discectomy have been reported in the literature. Therefore, this article aims to present a case of DPOSEH following OSE discectomy, and to provide a discussion on this potentially devastating complication.

## Case report

A 69-year-old male was admitted from the emergency service for evaluation of worsening right lower limb pain. He reported a one-month history of insidious-onset low back pain with associated radicular symptoms down the right leg, which had acutely intensified over three days. The pain, localized to the anterior thigh and anterolateral calf, was unresponsive to conservative measures and had escalated in intensity. The patient's medical history was notable for an allergic constitution and a 40-year smoking history, without any record of hypertension, diabetes, cardiac, cerebrovascular, or infectious diseases. Upon admission, his blood pressure was 130/90 mmHg. Laboratory tests showed Rh-positive blood type, a platelet count of 321 × 10^9^/L (reference range: 125–350 × 10^9^/L), a prothrombin time of 12.60 s (reference range: 10.7–14 s), an activated partial thromboplastin time of 36.74 s (reference range: 21–35 s), and an International Normalized Ratio (INR) of 1.05 (reference range: 0.8–1.2). Routine blood tests, liver and kidney function, electrolytes, and other laboratory investigations showed no significant abnormalities. The physical examination on admission revealed spinal percussion tenderness in the lumbar region. Sensation to pinprick was intact in the saddle area, and the anal reflex was present. Compared to the contralateral side, pinprick sensation was heightened over the anteromedial skin of the right lower limb. Manual Muscle Testing (MMT) demonstrated grade 5 strength with normal tone in all four limbs, and pathological signs were absent. The right straight leg raise and femoral nerve stretch tests were positive, while both patellar and Achilles tendon reflexes were normal.

**Figure 1:**
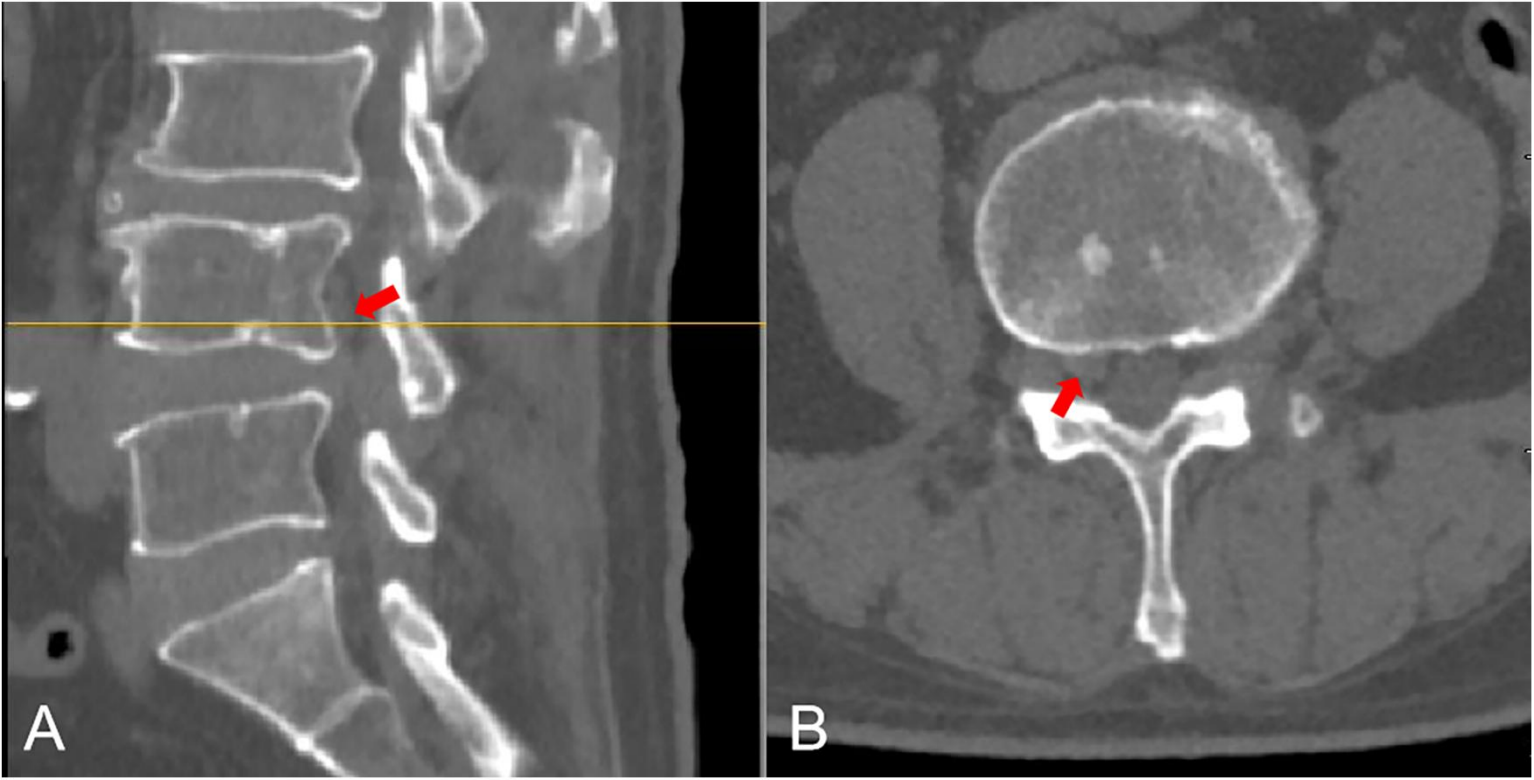
Preoperative CT revealed an L4/5 disc extrusion (indicated by a red arrow); **(A)** L4/5 disc extrusion had migrated superiorly. **(B)** Axial image demonstrated a significant right-sided disc herniation at the L4/5 level.

**Figure 2:**
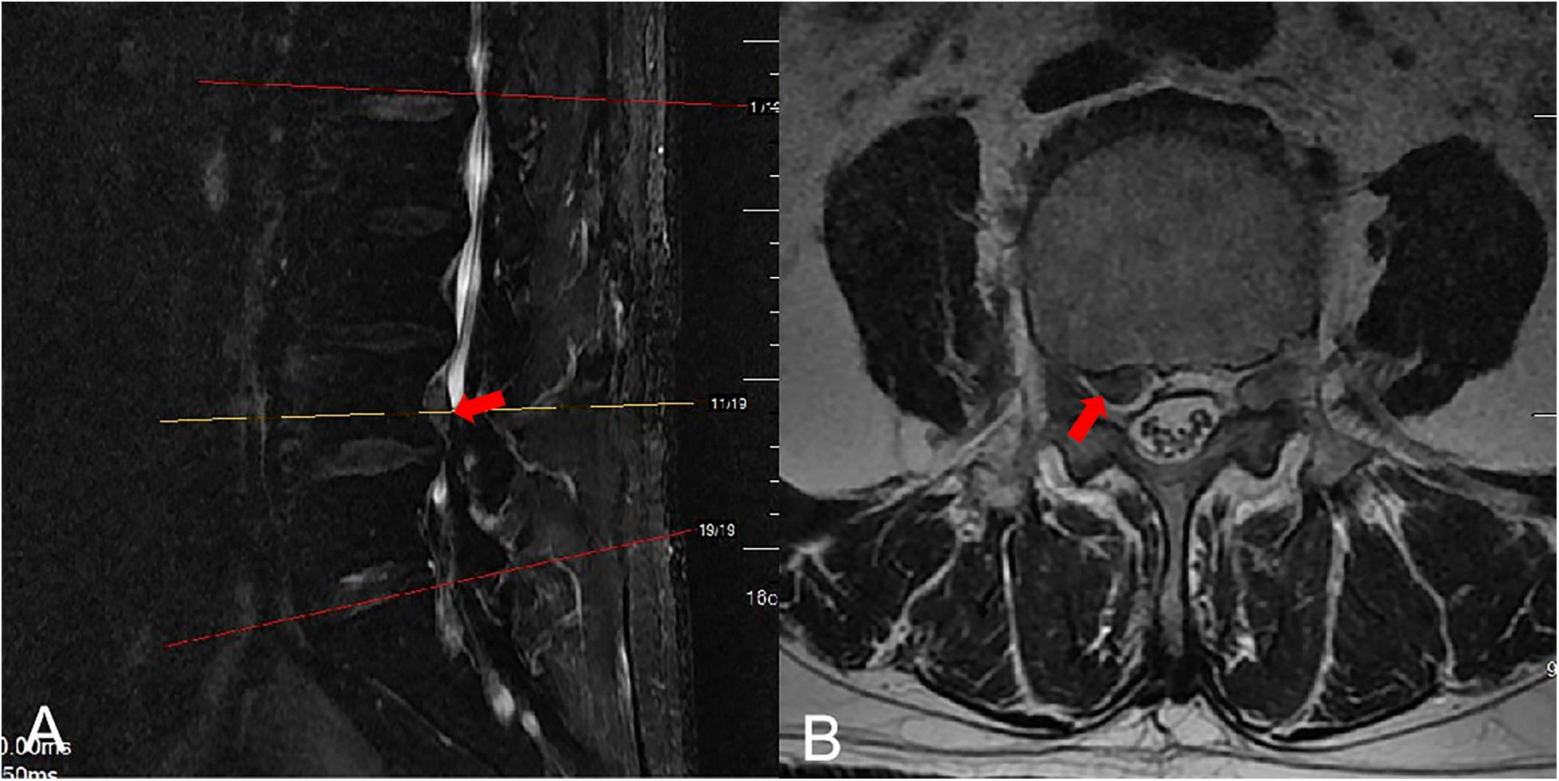
Preoperative MRI of the herniated disc. **(A)** Sagittal image demonstrated an extruded and sequestered L4/5 disc fragment (red arrow) migrating upward behind L4. **(B)** Axial image demonstrated the right L4 nerve root is encroached by the herniation (arrow indicates the extruded nucleus pulposus).

**Figure 3:**
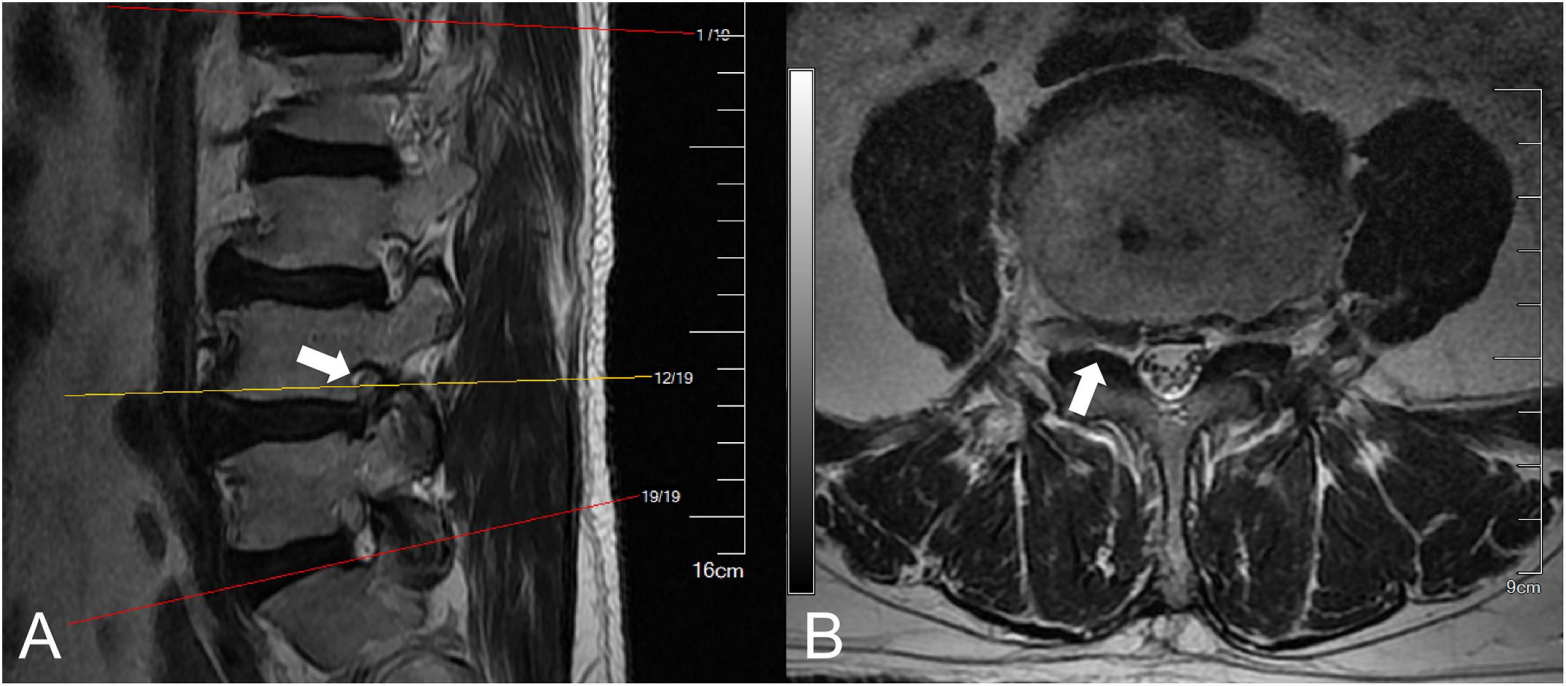
MRI views of the right L4/5 neural foramen. **(A)** Sagittal and **(B)** axial images demonstrated moderate foraminal stenosis. The exiting L4 nerve root is partially surrounded by epidural fat (white arrows), and there is no observable deformation of the nerve root.

Preoperative CT showed a large disc extrusion at L4/5 (Figure 1). Lumbar MRI on admission suggested a possible L4/5 disc herniation with sequestration and a free fragment. Right-sided stenosis was noted at the L4–L5 level (Figure 2, 3).

The patient was diagnosed with lumbar disc herniation with radiculopathy (L4/5). After excluding surgical contraindications, the patient underwent One-Hole Split Endoscopic spinal canal decompression and discectomy under general anesthesia on the 4th day after admission. Intraoperative confirmation indicated adequate decompression of the L4 and L5 nerve roots. A negative-pressure drain was placed postoperatively, with the procedure completed uneventfully. The drain output was 25 mL within the first 24 h and negligible on postoperative day 2. No postoperative anticoagulants were administered during this period.

From postoperative days 1–4, the patient exhibited reported constipation but exhibited marked improvement in lower limb pain and numbness, with no sensory or motor deficits reported. Superficial sensation remained intact, and muscle strength was preserved at grade 5 bilaterally. Laboratory studies demonstrated no significant coagulation abnormalities. The drainage tube was removed on day 2, and the patient began protected ambulation. On the night of postoperative day 5, the patient developed fecal incontinence, light red urine, and deep incisional back pain, accompanied by persistent bilateral lower limb pain and left-sided weakness. Physical examination revealed decreased sensation in the perianal skin and saddle area. MMT of the bilateral quadriceps, tibialis anterior, extensor hallucis longus, and gastrocnemius muscles was approximately grade 1. Both knee and Achilles reflexes were diminished. Straight leg raise and femoral nerve stretch tests were negative, and no pathological signs were observed.

**Figure 4:**
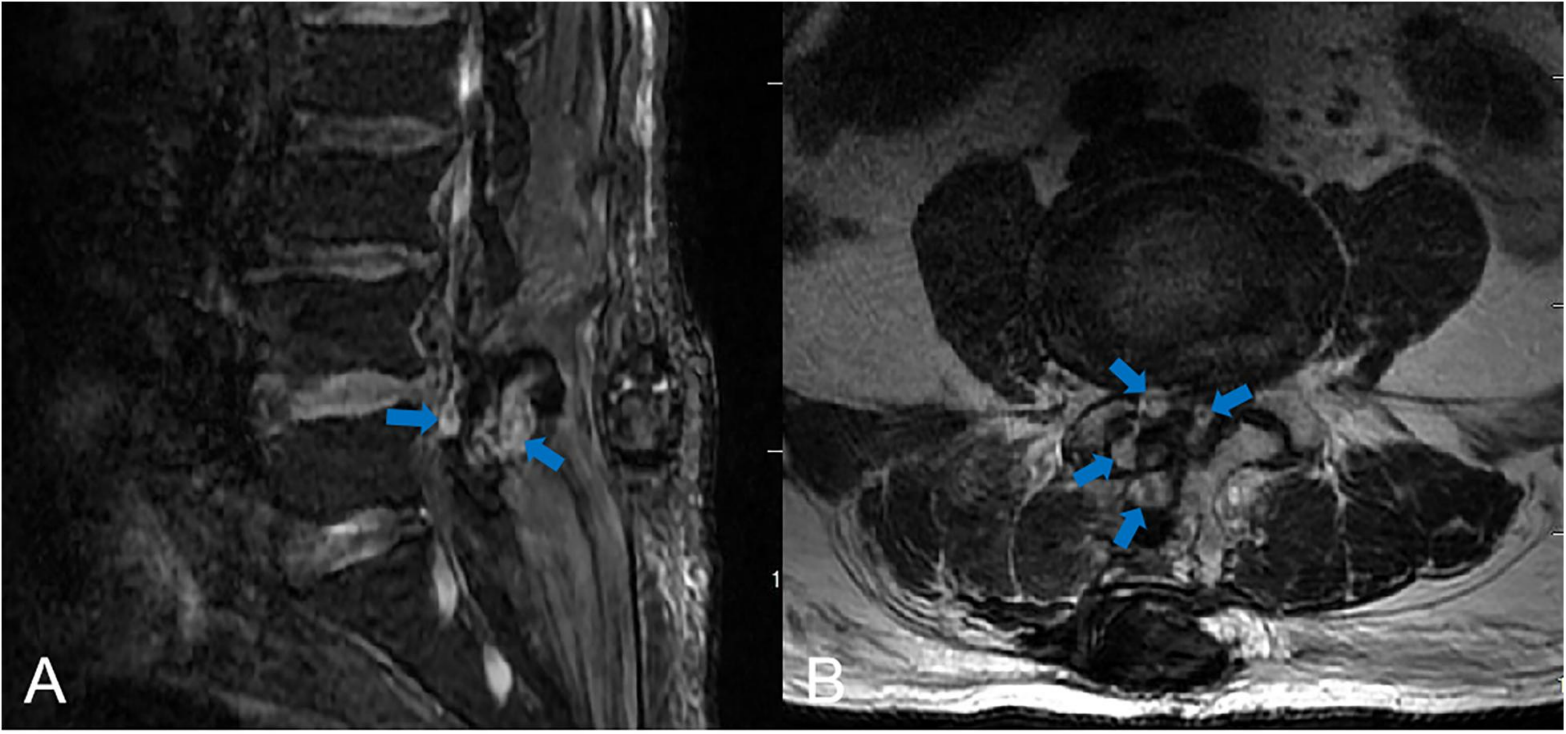
Postoperative MRI revealed a lumbar postoperative epidural hematoma. **(A)** Sagittal and **(B)** axial images demonstrated that in this fat-suppressed T2-weighted sequence, the epidural hematoma appears hyperintense (indicated by blue arrows).

Based on emergency lumbar MRI findings of multiple hyperintense epidural hematomas compressing the thecal sac (Figure 4), we performed emergency surgical evacuation for the resulting cauda equina syndrome. The procedure revealed multiple hematomas of varying sizes, with no active bleeding observed. Decompression was achieved by removing the L4 spinous process and partial laminae, followed by clot evacuation and control of bleeding with bipolar electrocautery. All nerve roots were then thoroughly explored and released, followed by irrigation and achieving complete hemostasis.

In the patient's postoperative rehabilitation, in addition to conventional anti-inflammatory analgesic and corticosteroid therapy, we administered acupuncture, functional electrical stimulation of the bladder area, infrared thermotherapy, and neuromuscular electrical stimulation. The patient was also guided to perform active ambulation with a walker device.

On examination within 15 days after hematoma evacuation, the patient demonstrated the absence of voluntary sensation for bowel movements and required indwelling catheterization for urination. Sensory examination revealed decreased sensation over the left perianal skin. Muscle strength assessment showed approximately Grade 4 strength in key muscles of the right lower limb, with left tibialis anterior and gastrocnemius muscles at Grade 3, and left extensor hallucis longus and extensor digitorum muscles at Grade 0. Following the comprehensive regimen, follow-up revealed a gradual improvement in muscle strength of the left extensor hallucis longus and extensor digitorum longus, which recovered to grade 1 by day 18, grade 2 by day 21, and grade 3 by day 45 after debridement. However, bowel and bladder function had not returned. At the 130-day postoperative follow-up during readmission for rehabilitation, examination showed that motor strength in the lower limbs had recovered to Grade 4 in major muscle groups, enabling independent walking. Anal sphincter tone demonstrated partial recovery with voluntary contraction. However, significant bowel and bladder dysfunction persisted: the patient reported awareness of rectal fullness but inadequate expulsion force and remained dependent on an indwelling urinary catheter.

## Discussion and review

### Clinical presentation and diagnostic confirmation

Our case represents a classic presentation of DPOSEH with initially favorable recovery followed by delayed neurological deterioration. Our patient exhibited acute neurological decline on postoperative day 5, featuring deep back pain at the incision site, new-onset flaccid paraparesis (muscle strength ∼1/5), saddle anesthesia, and bowel/bladder dysfunction. This constellation of symptoms constitutes the characteristic triad of cauda equina compression (10), with onset beyond 72 h confirming its delayed nature. MRI confirmed the diagnosis, revealing a hyperintense epidural mass from L4 to L5 causing significant compression on fat-suppressed T2-weighted imaging. Surgical exploration directly corroborated the MRI findings. The procedure revealed the presence of several substantial compressive clots within the epidural space.

### Mechanism of progressive venous epidural hematoma

The risk of bleeding in MISS is often underestimated compared to open procedures. While extensive decompressions and instrumented fusions are traditionally considered to predispose to epidural bleeding due to a larger raw surface area and greater venous disruption (10), data paradoxically indicate a higher incidence of symptomatic POSEH following MISS (11–14). A meta-analysis found the pooled incidence to be approximately five times higher with minimally invasive approaches (∼1.94%) than with traditional open surgeries (∼0.42%) (11). Considering the characteristics of OSE, a plausible explanation for this paradox is that continuous irrigation and limited visualization make minute bleeding points difficult to detect. The most likely scenario is that a small venous bleed persisted postoperatively at a very slow rate. A tiny laceration or oozing point in the epidural venous plexus might have been present. Over several days, this slow accumulation of blood could form a substantial hematoma (15).

Inadequate postoperative drainage is a possible contributing factor to the delayed hematoma in this case. Insufficient drainage is a recognized risk factor for hematoma formation, as unevacuated residual bleeding can accumulate within the closed surgical space and eventually form a significant hematoma (16). For reference, the average drain output after single-level discectomy is approximately 72 mL over three days (17). Our patient's output of 25 mL on the first postoperative day was slightly below this average and ceased completely by day 2, which does not preclude the possibility of inadequate postoperative drainage. Interestingly, the role of surgical drains in prevention remains debated, some studies associate premature removal or omission of drains in extensive surgeries with a higher risk of hematoma (18–20), while others find their use does not significantly reduce the incidence (21, 22). In practice, many surgeons leave a drain for 24–48 h in most cases and ensure it remains functional. The drain should not be removed too early if output is significant.

In the present case, the patient's age of 69 years is a likely contributing factor to the bleeding. Advanced age, typically defined as over 60 years, is another significant risk factor (23). Age-related anatomical and physiological changes, such as reduced epidural space compliance, spinal stenosis, and a higher incidence of comorbidities like hypertension, can all promote bleeding.

Another possible contributing factor involves the patient's early ambulation and constipation, both of which could induce a Valsalva maneuver and increase intra-abdominal pressure. This is a potential risk factor because the valveless, thin-walled epidural venous plexus is highly sensitive to pressure fluctuations (24). A sudden rise in intra-abdominal or intrathoracic pressure can transmit directly into this plexus, potentially rupturing fragile veins or dislodging an initial thrombus, leading to renewed bleeding and hematoma enlargement even days after surgery.

Alternatively, a venous congestion vicious cycle may have contributed. The established epidural hematoma compresses the venous plexus, impeding venous outflow. The resulting venous congestion raises local venous pressure further in a vicious cycle, promoting more bleeding into the epidural space. Epidural veins are thin-walled and bleed easily when distended, making continued or intermittent bleeding around the clot possible, which rapidly enlarges the hematoma (25).

### Factors limiting neurological recovery

The timing of emergent surgical decompression is critical for optimizing neurological outcomes. In this case, the unavoidable delays from analgesic observation, urgent MRI confirmation, and preoperative consent discussions contributed to a postponed intervention, which is a significant factor in the patient's residual neurogenic bladder and moderate lower limb weakness. Earlier decompression is associated with improved neurological recovery (26, 27), with the ideal “golden window” being within 6 h of symptom onset to offer the best chance for complete functional restoration (8). One study found that patients decompressed within 6 h of deficit onset improved by an average of 2 Frankel grades, compared to only ∼1 grade for those decompressed later (7). Conversely, full recovery is rare after 72 h of ongoing compression (28). Therefore, decompression should occur as soon as humanly possible once the diagnosis is made.

By the time decompression was performed, the cauda equina had likely been under pressure for several hours, with overt sphincter dysfunction already present, potentially leading to irreversible axonal degeneration or neuronal cell death (16). In patients with postoperative spinal epidural hematomas, those with milder deficits often recover fully, whereas those presenting with complete paralysis or loss of sphincter function have a higher likelihood of permanent deficit (9, 15). One study reported that among patients with delayed spinal epidural hematomas, some had persistent neurological deficits postoperatively; in their series, 2 of 7 patients did not fully recover (15). Similarly, a comparative study documented a few cases with “poor” neurological outcome despite hematoma evacuation (9). These data indicate that the extensive neural damage already present at the time of intervention limits the potential for complete functional recovery.

Additionally, one study suggests a potential reperfusion mechanism underlying this process (15). Following hematoma evacuation, reperfusion may paradoxically induce further inflammation or free-radical damage, which compounds the initial metabolic insult and explains incomplete neurological recovery despite surgical decompression.

### Additional risk factors reported in literature

Patient-related factors significantly influence the risk of postoperative spinal epidural hematoma (POSEH). Major risk factors include coagulation disorders or anticoagulation therapy (reported in approximately 44% of cases (10, 28). Furthermore, postoperative hypertension is a well-recognized risk factor, with studies showing an association between acute blood pressure spikes and hematoma formation (29, 30). Other comorbidities, including hepatic or renal dysfunction, chronic alcohol use, and hematologic disorders such as thrombocytopenia, can further increase the risk (26, 29, 30). Some evidence also suggests that diabetes mellitus and obesity may be associated with a higher incidence of POSEH, possibly due to more extensive degenerative disease and altered healing processes (30).

Surgical factors also contribute to the risk of POSEH. Larger and more invasive procedures inherently carry a higher risk due to greater tissue dissection and longer operative duration, which can lead to increased blood loss and more opportunity for bleeding to accumulate. One study identified operating on more than five spinal levels and intraoperative blood loss exceeding 1 L as significant risk factors for epidural hematoma (23). Prior surgery at the same level is also considered a predisposing condition, as epidural scar tissue may reduce the space available for tamponade and increase vascular fragility during dissection (28). Additionally, incidental durotomy with cerebrospinal fluid (CSF) leakage is a recognized risk factor in some series (16). A CSF leak can lower intrathecal pressure, potentially leading to intracranial hypotension and engorgement of the epidural veins, thereby promoting bleeding.

It is noteworthy that while a clear link to the aforementioned risk factors was not established in the present case, the literature unequivocally confirms their existence. These factors remain essential considerations for surgeons and warrant vigilant clinical awareness.

### Surgical and conservative management

The presence of a symptomatic POSEH is a surgical emergency. Urgent surgical decompression usually involves reopening the prior incision, performing a laminectomy, or expanding the existing one if needed, and evacuating the clot to relieve pressure on the neural elements. Any active bleeding sources are cauterized or controlled, and the wound is thoroughly hemostatic before reclosure.

There are several reports of conservative management. The administration of high-dose corticosteroids represents a controversial non-surgical option in the management of spinal epidural hematomas. The consensus is that this is rare and only appropriate in highly selected cases. Ghaly and Cuenca reported rapid symptomatic improvement after intravenous methylprednisolone in cervical and thoracic cases, respectively (31, 32). Lin et al. (33) documented one successful and one failed dexamethasone trial in traumatic cervical hematomas, underscoring the unpredictability of this approach. Importantly, non-operative management is only feasible when neurological deficits are minimal and stable. However, given that our patient presented with significant neurological deficits, emergent surgical intervention was the only reasonable course of action.

## Conclusion

In conclusion, we present a case of delayed POSEH following OSE surgery, which, to our knowledge, has not been described before and serves to alert the surgical community. This unique occurrence underscores a critical clinical reminder: the risk of delayed hemorrhage, while uncommon, is a potential threat shared across the spectrum of spinal procedures, including novel minimally invasive techniques. Consequently, maintaining a high index of clinical suspicion, ensuring vigilance during the postoperative period, and preparing for rapid diagnostic and surgical intervention remain paramount to optimizing patient safety and long-term prognosis.

## Data Availability

The original contributions presented in the study are included in the article/Supplementary Material, further inquiries can be directed to the corresponding authors.
